# Unrecognized importance of hawking prey capture tactics in a tropical plunge-diving seabird

**DOI:** 10.1098/rsbl.2026.0292

**Published:** 2026-11-04

**Authors:** Abigail W. Schiffmiller, Charles A. Braman, Michael Johns, Sara Maxwell, Hillary S. Young, Scott A. Shaffer, Greg A. Breed

**Affiliations:** ^1^ Department of Biology and Wildlife, University of Alaska Fairbanks, Fairbanks, AK, USA; ^2^ Institute of Arctic Biology, University of Alaska Fairbanks, Fairbanks, AK, USA; ^3^ Department of Ecology, Evolution and Marine Biology, UC Santa Barbara, Santa Barbara, CA, USA; ^4^ Point Blue Conservation Science, Petaluma, CA, USA; ^5^ University of Washington Bothell, Bothell, WA, USA; ^6^ Department of Biological Sciences, San Jose State University, San Jose, CA, USA

**Keywords:** aerial capture, aerial pursuit, facilitated foraging, *Exocoetidae*, *Ommastrephidae*, biologging, foraging behaviour, *Sula sula*

## Abstract

Seabirds are critical ecosystem members providing connectivity between marine and terrestrial ecosystems. Seabird foraging is often facilitated by other marine predators (e.g. tuna) that aggregate prey at the surface. Some seabirds have been anecdotally reported to ‘hawk’ (catch mid-air/in-flight) flyingfish and flying squid forced into flight by subsurface predators. Hawking avoids water entry and subsequent take-off, both of which are known to be energy intensive, but its relative importance in foraging has not been quantified. We analysed footage from 25 animal-borne video cameras deployed on red-footed boobies (*Sula sula*) at Palmyra Atoll to estimate the frequency of hawking, plunge diving and ‘other’ prey capture attempts. Hawking made up 20.3% of all prey capture attempts and had a 39.3% success rate in 2022, but was not observed for the two tracked birds in 2024. An additional 17 individuals were opportunistically observed in the footage making hawking capture attempts, suggesting hawking is a common behaviour, accounting for a greater proportion of foraging effort than previously known. As hawking is only possible when subsurface predators elicit flight escape behaviours of prey, its high frequency suggests such predator facilitation interactions are stronger than currently understood.

## 1. Introduction

Seabirds are integral members of pelagic, nearshore and terrestrial ecosystems and abundant mid- and upper-trophic-level predators, with global estimates in the hundreds of millions of individuals [1]. However, they are also among the most threatened, with nearly half of species in decline, 35% listed as threatened or near-threatened [2] and global seabird abundance probably less than 30% of historical averages [1]. In the tropics, plunge-diving sulids (boobies) are some of the most common seabirds and provide the largest nitrogen and phosphorus subsidies, key in supporting reef ecosystem health [3,4], per breeding colony at atolls across the globe [5].

Though known as plunge-diving specialists, boobies have been anecdotally observed to pursue, catch and swallow airborne prey in flight [6–12] (hereafter ‘hawking’ for simplicity). Similar foraging tactics (e.g. ‘air dipping’ [13] and aerial pursuit [14]) are also frequently reported for smaller species, including terns [13,15,16]. However, the frequency of hawking behaviour, its success rate relative to typical sulid plunge diving tactics, and its overall importance to foraging efficiency is essentially unknown. Hawking avoids energetically costly behaviours associated with plunge diving, as it does not incur the high-G impact with the water surface nor require take-off to continue foraging [17–19], and therefore may be an important foraging tactic if common.

Critically, for birds targeting marine prey, hawking is naturally only possible when foraging is facilitated by other marine predators—such as tuna, dolphins and sharks—which trap schools of prey against the surface and, in the case of flyingfish (*Exocoetidae*) and flying squid (*Ommastrephidae*), elicit their flight escape behaviour [20]. So important is this phenomenon that many tropical seabirds have become near-exclusive specialists on flyingfish and flying squid [13,20–23] and in some cases are considered nearly ‘tuna-obligate’ [16,20,22]. If seabirds are heavily reliant on hawking, and specifically predator facilitation-driven hawking, particularly during the breeding season when they are central place foragers, they may be more vulnerable to climate- or fishing-pressure–driven shifts in prey and marine predator populations than previously recognized [16]. Although, especially in more anthropogenically impacted systems, some birds also take advantage of ‘vessel facilitation’ to capture air-borne prey [9,24], these opportunities are probably less reliable near most breeding locations. If hawking is more common than previously known for some of these species, failing to account for it in estimates of foraging effort could result in a misunderstanding of true foraging effort and an underestimate of the importance of predator facilitation interactions to seabirds.

Biologging may offer a solution to understanding the importance of hawking behaviour and its relationship to subsurface predators. Biologgers, including accelerometers, temperature-depth recorders (TDRs) and wet–dry sensors, have been used to identify foraging bouts and quantify foraging effort of free-living sulids [7,11,19,25–27]. However, these studies probably miss hawking behaviour because it is not readily identifiable from the resulting data. Animal-borne cameras offer an opportunity to address this gap. As cameras have become smaller and more battery-efficient [28–32], animal-borne video has become a viable way to observe animal behaviours when direct observations are not possible. Animal-borne video footage can be used to assess the relative frequency and importance of behaviours like hawking, and potentially can be used to identify them in other biologger data in future research.

Red-footed boobies (*Sula sula*; abbreviated ‘RFBO’) are the smallest [33] and most pelagic of boobies [34] and one of the most globally abundant sulids [2]. They are widely reported to forage in association with marine predators and consume primarily flyingfish and flying squid, with many anecdotal reports of in-flight captures [6–9,11,20,24]. Here, we deployed animal-borne cameras, with GPS and accelerometry biologgers, on nesting RFBO at Palmyra Atoll (a small atoll in the Central Tropical Pacific), to estimate the contribution of hawking foraging tactics to overall foraging effort. We show that hawking behaviour is quite common, with a high capture success rate for RFBO.

## 2. Material and methods

### (a) Data collection

Video cameras, GPS loggers and accelerometers were deployed on RFBO at Palmyra Atoll (5.88° N, 162.07° W) in May and November 2022, October 2023 and November 2024. All birds selected for tagging were incubating eggs or brooding young chicks; birds with older chicks were avoided because irregular nest attendance made tag recovery more difficult. Pairs in this nesting stage usually alternate day-long foraging trips with a day tending the nest, though observed foraging trips often ranged up to 2 or 3 days and rarely 4 or more. Only one mate from a pair was captured and tagged. The tracked individual was marked with a permanent marker or non-toxic nail polish on their breast and/or the top of their head for easy identification from a distance and to avoid unnecessary disturbance of their mate.

For both logger deployment and retrieval, adult RFBO were caught from their nests by hand or with a telescoping noose pole. Nests less than 2 m from the ground were considered in range, whereas nests at greater heights were avoided. During the initial capture, all birds were equipped with a unique USGS metal ID band. Mass was recorded for both deployment and retrieval captures by placing birds in a cloth bag and weighing with a spring balance (±20 g). Equipment deployed on each bird was adjusted to ensure the total mass was no greater than 3% of the bird's mass at deployment [35]. Loggers were attached to feathers with removable adhesive waterproof tape (Tesa tape 4651, Hamburg, Germany) and cyanoacrylate glue. Cameras (table 1) were attached facing forward to the central back contour feathers. Accelerometry and accelerometry/GPS, loggers (table 1) were attached to the back behind the camera, directly over the bird's centre of mass. Stand-alone GPS units (table 1) were attached to the three central tail feathers. Lighter animals were generally equipped only with GPS and accelerometers or occasionally with cameras and our lightest GPS unit. Cameras were not deployed on birds weighing less than 750 g.

**Table 1.** Biologger details: types, settings and attachment points.

logger type	manufacturer: model	mass (g)	settings	attachment location
camera	Little Leonardo: DVL400M	15	30 fps	central back contour feathers
accelerometer	Technosmart: Axy 5XS	3	25 Hz, 8 Gs	central back contour feathers, behind camera, directly over the bird's centre of mass
accelerometer + GPS	Technosmart: AxyTrek Mini	7.5	GPS: 30 s fix rate; Axy: 25 Hz, 8 Gs	central back contour feathers, behind camera, directly over the bird's centre of mass
GPS	Catlog: Gen2	12	30 s fix rate	central 3 tail feathers
GPS	Lotek: Pinpoint240	7.5	1 min fix rate	central 3 tail feathers

Cameras were programmed to turn on between 10 AM and noon local time the day following deployment to capture foraging behaviours when birds were farthest from the colony and most likely to be actively foraging [25]. Cameras were programmed to record until battery life was exhausted (roughly 4 hours) in a single day, or to record for 2 hours at the same time each day until the battery was exhausted. After tags were deployed, nests were checked every day until the tagged bird returned from its foraging trip (most often 48 hours after the tags were deployed). Tags were retrieved by recapturing the bird, and all tags and tape were removed. Deployments with successful retrievals typically lasted 2–4 days.

### (b) Video annotation and analysis

Videos were reviewed and annotated using Behavioural Observation Research Interactive Software (BORIS) v.8.20.3 [36]. Each video's unobscured sections were reviewed at half speed or slower at least twice. Annotations were made for a wide suite of behaviours and conditions, and all prey capture attempts (hereafter ‘attacks’) were counted and categorized.

Attacks were categorized as ‘plunge dives’, ‘hawking’ and ‘other’ (figure 1; supplementary material, table S1, videos SV1–SV3). Plunge dives were characterized by a rapid flip or downward turn in flight and steep dive into the water from some height. Hawking was characterized by flight low over the water surface in pursuit of airborne prey without landing, regardless of capture success. Instances of near-horizontal water entry from low flight and of dipping dives from sitting on the surface as well as otherwise unclassified attacks were categorized as ‘other.’ Behaviours such as theft from other birds or scavenging were not counted as attacks. Aborted attacks were noted separately, though their identification is inherently subjective. Aborted plunge dives were counted when the bird was deemed to have initiated a dive but resumed level or ascending flight without reaching the water surface, and aborted hawking was counted when the bird soared close to the water surface behind airborne prey, but was deemed not to have targeted an individual for attempted capture.

**Figure 1.**
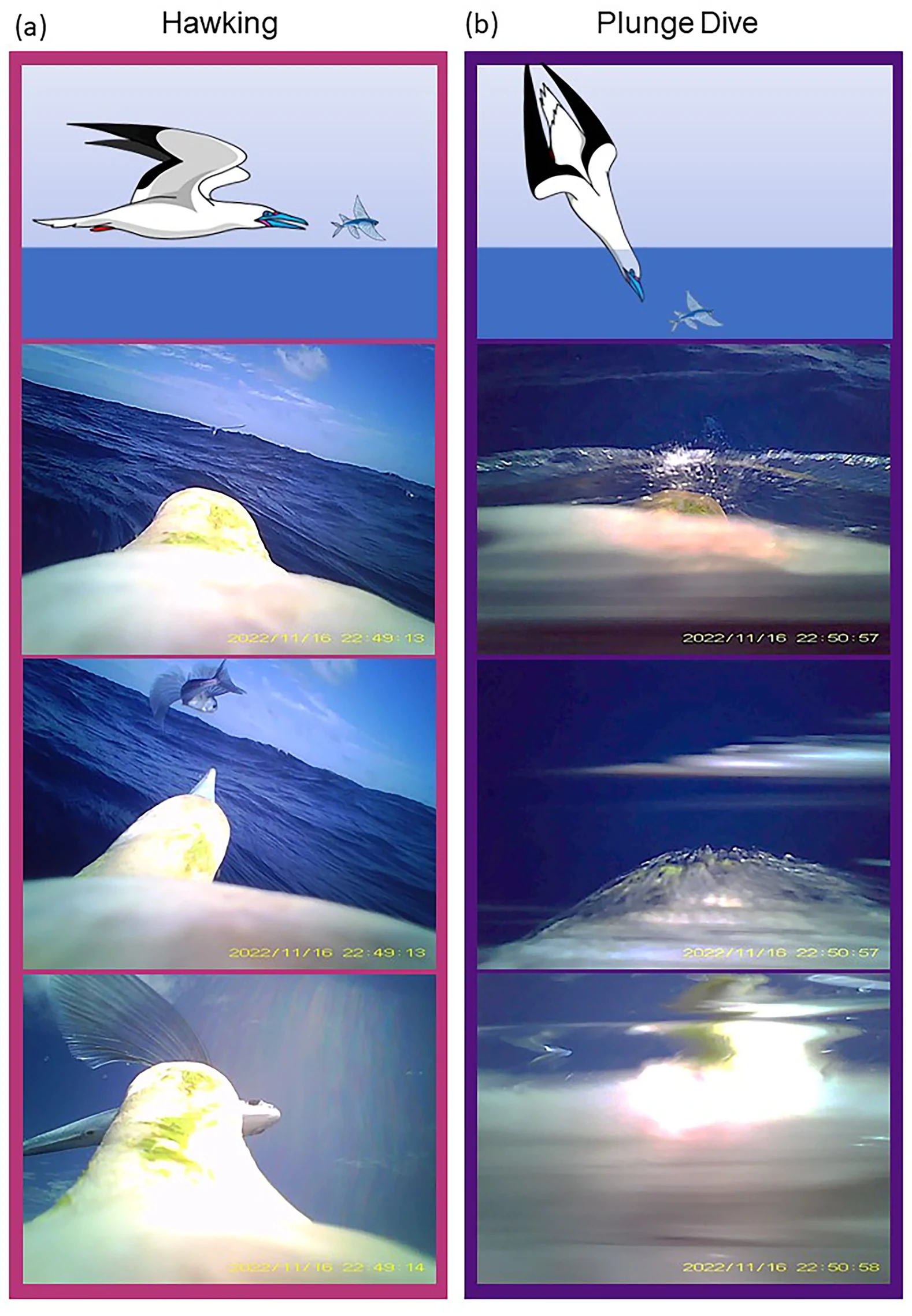
Examples of hawking and plunge diving from video footage. (a) Diagram and sequence of three still images from video footage of hawking attack (successful). (b) Diagram and sequence of three still images from video footage of plunge dive attack (success unknown). Time stamps in UTC (local time UTC-10). See supplementary material, videos SV1 and SV2.

Each attack was categorized as successful, failed or unknown. An attack was counted as successful if it was clear the bird had caught prey in its bill, even if swallowing could not be confirmed (figure 1a; supplementary material, videos SV1 and SV2). Attacks were counted as failed if prey was not caught (supplementary material, videos SV1 and SV2). Attack success was counted as unknown when footage was obscured by water or feathers and prey capture could not be confirmed or ruled out (figure 1b; supplementary material, video SV1). Aborted plunge dives and hawking attempts were counted only as ‘aborted.’

To calculate attack rate per minute overall and for each attack type, attack counts were divided by the time between the first and last observed attack, including aborted attacks, plus one minute on either end. This was done to exclude periods when birds were commuting between the nest and foraging areas from attack rate calculations. This ‘observed attack window’ included any time when the camera footage was obscured and when birds may have been traveling between foraging bouts.

Attacks made by other untagged birds (individuals not carrying biologging equipment but captured on video by camera-tagged individuals) were noted when enough of the attack was in frame to categorize the type (supplementary material, figure S1A and video SV4).Success was determined in the same manner as for attacks by tagged birds. However, these events were not included in calculations for prevalence of attack types or rates as they were observed opportunistically.

## 3. Results

Video cameras were deployed on 47 individuals at Palmyra Atoll (33 in 2022, 4 in 2023 and 11 in 2024), and 36.7 hours of unobscured footage was recovered and analysed from 25 individuals. We note that 2023 was an El Niño year with low nest numbers and a large degree of nest failures, and during the 2024 field season the number of nesting birds was still a tiny fraction of that observed in 2022 and was still recovering from the 2023 El Niño event. Usable footage was not retrieved from any birds in 2023, and from only two birds in 2024. A total of 357 completed attacks and 98 aborted attacks made by tagged birds were identified. Total attack counts (excluding aborted attacks) for individuals ranged from 1 to 49, and hawking attacks accounted for 0%–100% (mean = 19.5%) of observed attacks for any given individual (figure 2). Total attacks per minute over the observed attack window ranged from 0.16 to 2.06 (mean = 0.78).

### (a) Hawking attacks

Of the 25 tagged individuals with attacks captured on camera, 20 made at least one hawking attack (figure 2), indicating that it is a normal and regular foraging behaviour. Hawking attacks accounted for 20.3% of all 2022 attacks (figure 3). Success or failure of hawking attacks was easy to determine as the field of view remained largely or fully unobscured, and 39.3% were successful (figure 3; supplementary material, video SV2). In addition, 16 hawking attacks (9 successful) made by birds near a camera-carrying bird were opportunistically observed and recorded in 2022 (supplementary material, figure S1A and video SV4). In the 2024 footage, no hawking attacks by either camera-tagged bird were observed, despite one having the greatest total number of observed attacks in our dataset (49; figure 2), but a single successful hawking attack by another RFBO was observed. Hawking attack rate ranged from 0 to 0.52 (mean = 0.09) attacks per minute over the observed attack window when all birds were included and from 0.03 to 0.52 (mean = 0.13) when only birds with at least one hawking attack were included.

**Figure 2.**
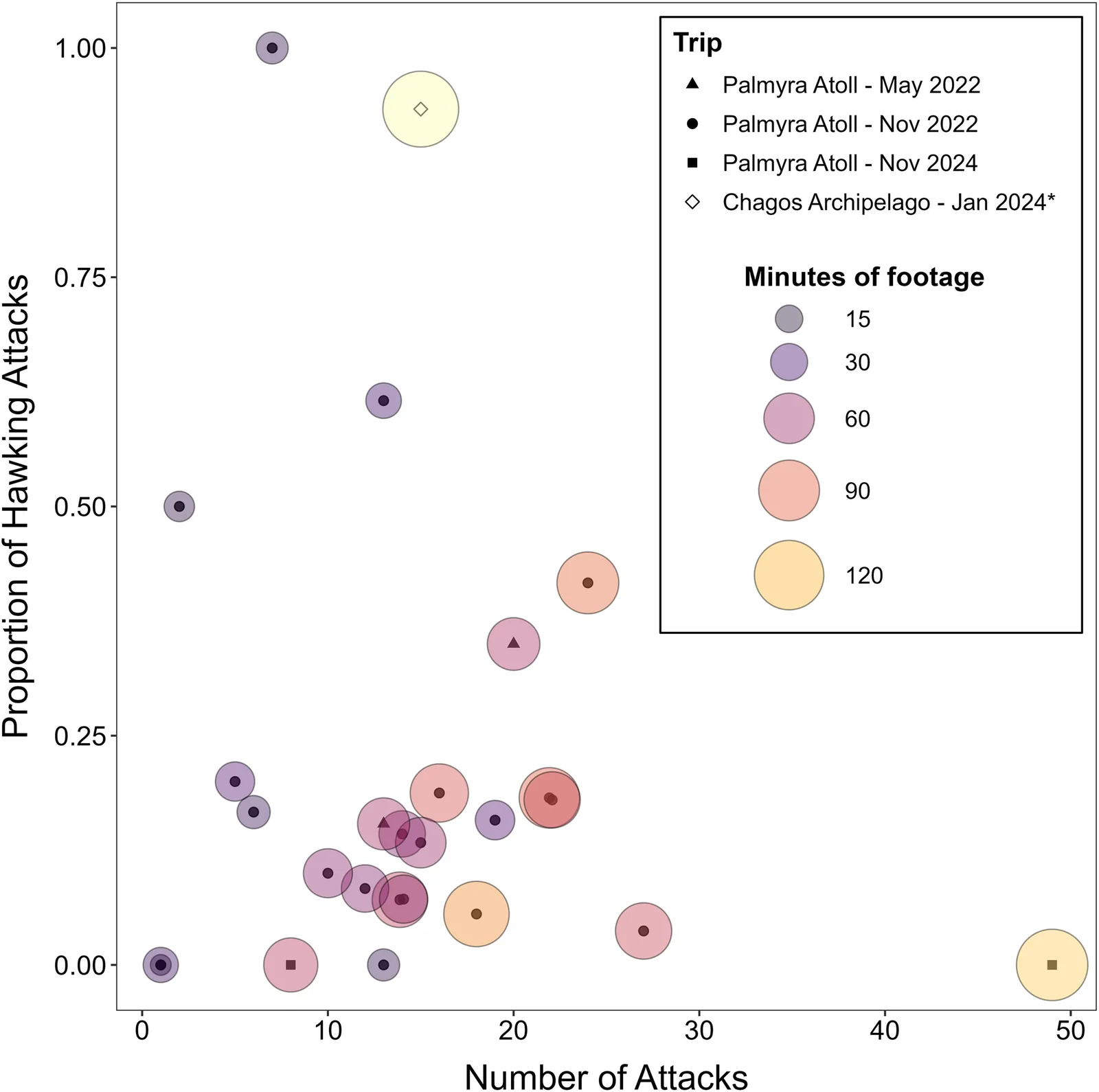
High variation in proportion of hawking, number of observed attacks and total minutes of unobscured footage per bird. Palmyra Atoll November 2024 birds not included in counts in figure 3 (see §4). *Chagos Archipelago data as reported by [11].

**Figure 3.**
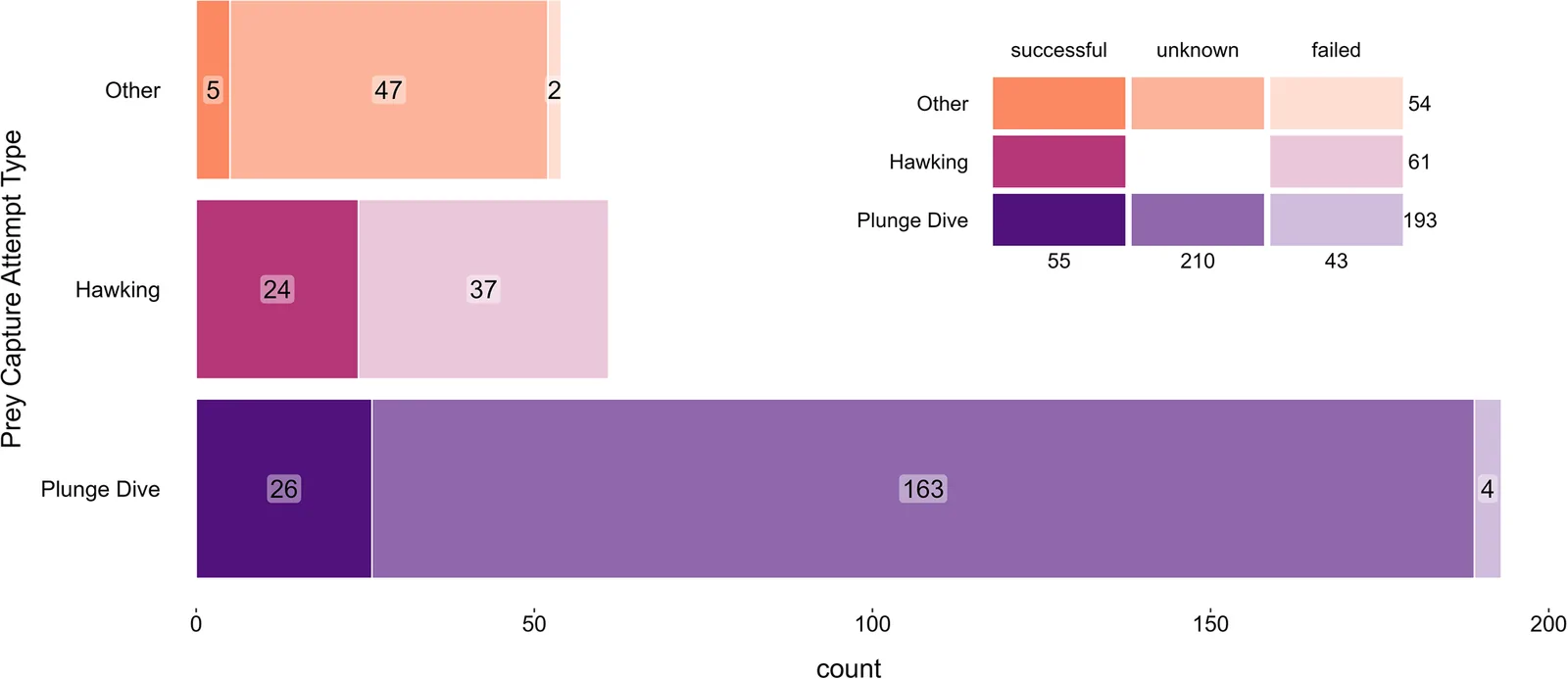
Attack type and success counts, 2022. No hawking attacks had unknown success. Aborted plunge and hawking attacks are not included. Opportunistically observed attacks made by other individuals are not included.

### (b) Plunge-diving attacks

Of all attacks in 2022, 64.1% were plunge dives (figure 3). Success could not be determined and was unknown for the majority of plunge dive attacks (84.5%; figure 3) as the field of view was usually obscured by feathers, water and bubbles as the bird impacted, entered, sat on or exited the water (supplementary material, video SV1). Success was confirmed for 13.5%, and failure for only 2.1%, of plunge dives (figure 3). Between both 2024 birds, 47 plunge diving attacks (4 successful) were observed. Plunge diving attack rate ranged from 0 to 1.39 (mean = 0.4) attacks per minute.

### (c) Other and aborted attacks

The remaining 15.6% of all 2022 attacks were categorized as ‘other’ (figure 3). Of these, 55.3% were extremely shallow angle dives (as opposed to plunge dives initiated from a height; supplementary material, video SV3). At least one of these appeared to be an attempted hawking attack, but the bird incidentally struck the water as the fish it pursued re-entered the water. Underwater dives, starting from a position sitting on the surface, accounted for 34% of ‘other’ attacks. In 2024, nine ‘other’ attacks (all unknown success) were counted. Attack rate for ‘other’ attacks ranged from 0 to 0.39 (mean = 0.11) attacks per minute.

Eighty aborted attacks were noted in 2022. Of these, 55 (68.75%) were aborted plunge dives and 25 (31.25%) were aborted hawking attacks. In 2024, 16 aborted plunge dives and 2 aborted hawking attacks were identified. The aborted attack rate ranged from 0 to 0.65 (mean = 0.17) attacks per minute over the observed attack window. Across all 2022 and 2024 data, eight instances of scavenging, fighting with conspecifics over food, and theft from conspecifics were observed but not counted as attacks.

### (d) Prey

For two of the successful hawking attacks and one of the plunge diving attacks of unknown outcome, the targeted prey was flying squid (family *Ommastrephidae*; supplementary material, figure S1C,D and video SV5). For all remaining attacks where prey type could be identified, the targeted prey was flyingfish (family *Exocoetidae*, genus unknown). We noted many instances of flyingfish leaving the water in groups of tens to hundreds of individuals. These mass flights usually occurred repeatedly over the course of several minutes, and typically included evidence of marine predator presence. During these ‘fish storms’ (minimum *n* = 6) birds would often make multiple hawking attacks in sequence (supplementary material, figure S1B and video SV6). A total of 39 hawking attacks, nearly a third of those observed (19 failed, 9 successful, 11 aborted), made by our tagged birds (as well as 5 made by other birds) were observed in association with ‘fish storms’.

## 4. Discussion

Hawking accounted for 20% of attacks observed in animal-borne video footage and was successful 39% of the time, representing a substantial proportion of foraging effort for RFBOs. Though anecdotally reported, hawking behaviour has not been accurately accounted for in previous foraging descriptions for sulids. The prevalence of hawking attacks presented here indicates that a large proportion of RFBO foraging effort is not currently being identified using standard biologging analysis methods. For instance, using accelerometry, TDRs and wet/dry activity sensors, Weimerskirch *et al.* [7] reported that 93% of RFBO dives were plunge dives, and 7% surface dives initiated while sitting on the water. For the plunge dives, approximately 30% were described as ‘aerial’ dives that never entered the water. These proportions are roughly similar to the fraction of plunge dives that struck the water versus those that were aborted in our study. They proposed that the aerial dives ‘probably correspond to their specific prey capture technique [and] allow red-footed boobies to pursue and capture mobile prey such as flying fishes and squids at or above the surface’. However, our video footage indicates that these previously reported ‘aerial’ dives were instead most likely to have been aborted plunge dives, whereas hawking captures probably went undetected. Although a unique and consistent accelerometry signal may be discernible for hawking attacks, no studies have done so to date.

Although our study did not permit a good estimate of success for plunge dives, to compare against the success of hawking, others have shown success rates to be high. A 56% success rate was found for masked booby (*Sula dactylatra*) plunge dives (*n* = 9) [32]. For Australasian gannets (*Morus serrator*), Machovsky-Capuska and colleagues [31,37] found success rates of 43–45.5% for plunging dives without pursuit, and Cansse [38] reported a 57% success rate (with 20% unknown) for plunge-pursuit diving. Though RFBO perform shallower plunge dives [7,39,40] and engage in underwater pursuit comparatively rarely [40], their plunge dive success rates are expected to be similarly high. Plunge diving is an energetically costly method of foraging owing to the large amount of time spent in active flight and the necessity of energy-expensive take-offs [17,18] and regaining altitude to continue foraging, regardless of success. Although hawking also requires flight, it does not require a take-off and probably has a similar energetic cost to any other low-altitude flight, a common locomotor behaviour during foraging trips even when not actively foraging [12]. Hawking attacks, whether successful or not, impart almost no opportunity cost as the bird is immediately in position to make another attempt. The relative energetic consequences of hawking and plunge diving foraging tactics remain to be quantified, but hawking may increase overall foraging efficiency considerably, even if the energetic cost per unit time is similar or the success rate is lower.

The numerous documented examples of hawking attacks from the 25 RFBOs carrying cameras, and at least 17 other individuals opportunistically observed, demonstrate that this is a common behaviour for the Palmyra Atoll population of RFBOs. Data from other studies suggest that hawking is a species-wide behavioural trait and not a population-specific behaviour. Dunn *et al.* [11] reported multiple instances of hawking from a single camera-tagged RFBO, and additional conspecifics caught in frame, nesting in the Chagos archipelago in the Indian Ocean (figure 2), consistent with other anecdotal reports of RFBO hawking [6,8,9,41].

It is less clear if this behaviour is common across all RFBO individuals and conditions. Cameras only recorded a portion of each foraging trip, giving a small window into foraging during the breeding season. We found substantial individual variation in rates of different attack types (figure 2), indicating that individuals may be specializing in foraging tactics [42–44]. Similarly, the relative lack of hawking behaviour in 2024, when higher wind speeds were observed and ocean conditions were still relatively poor following the 2023 El Niño event, may be evidence that the use of hawking depends on local wind conditions, changes in prey populations or changes in subsurface predator populations driven by environmental variability (El Niño, marine heatwaves, ocean warming). The many observations of hawking by individuals unencumbered with tags support hawking as a normal behaviour, not one adopted only in response to the mass or placement of biologgers. Regardless of any differences in attack behaviours or rates owing to individual variation, environmental conditions or biologger attachment [45], the frequency of observed hawking attacks changes our understanding of foraging effort and behaviour for this species. Hawking may also be a more common behaviour than realized for other seabird species [10,15], and foraging effort may be considerably underestimated for them as well. By failing to account for hawking tactics in foraging by RFBO and other seabirds, foraging analyses may underestimate food intake, foraging efficiency, and the strength and importance of interactions between seabirds and other pelagic predators.

Many pelagic seabirds forage in aggregation with subsurface predators [21,22,41], increasing the efficiency of foraging by allowing repeated capture attempts and higher success rates within a reduced area and time [46]. Our study strongly suggests such facilitation interactions may be key to unlocking a separate and potentially highly efficient foraging tactic, hawking, for species traditionally considered to be plunge-diving specialists. This may require a re-examination of the foraging tactics of pelagic seabirds to quantify the contribution of this critical, but previously unexamined, behaviour. Although animal-borne cameras are key to documenting and validating it, they still have many challenges: heavy, short battery lives and recording windows, prone to being obscured by feathers and positioning, and requiring extreme effort to analyse and extract data. A critical next step will be using other biologging streams to identify and better understand this behaviour. Although very similar to skimming flight, a unique and consistent accelerometry signal may be discernible for hawking attacks, however no studies have described this signal to date. Accurately describing this signal and reassessing previous foraging data should be a priority for future work. It seems likely that marine-predator facilitated foraging will prove to be even more critical to these tropical seabirds than previously acknowledged, potentially changing how we think about what is needed to conserve these species.

## Supplementary Material



## Data Availability

Supplementary material is provided with the publication. Supplementary material [47], as well as code and video annotation files [48], and video data are also available from Zenodo [49,50]. Supplementary material is available online [51].
