# Supplementary material for: Unrecognized importance of hawking prey capture tactics in a tropical plunge-diving seabird
Source: Biol Lett. 2026 Nov 4;22(11):20260292. doi: 10.1098/rsbl.2026.0292 (PMC13730644; doi:10.1098/rsbl.2026.0292)
Supplement: Supplementary file 1 [file rsbl.2026.0292.zip › rsbl.2026.0292/rsbl20260292_si_001.pdf]

Unrecognized importance of hawking prey capture tactics in a tropical plunge diving seabird.

Abigail Schiffmiller[1*], Charles A. Braman[2], Michael Johns[3], Sara M. Maxwell[4], Hillary Young[2], Scott A. Shaffer[5], Greg A. Breed[1,6]

April 21, 2026

1. Department of Biology and Wildlife, University of Alaska Fairbanks, Fairbanks, AK, U.S.A.;

2. Department of Ecology, Evolution, and Marine Biology, University of California, Santa Barbara, CA, U.S.A.;

3. Point Blue Conservation Science, Petaluma, CA, U.S.A.;

4. School of Interdisciplinary Arts and Sciences, University of Washington, Bothell, WA, U.S.A.;

5. Biological Sciences, San Jose State University, San Jose, CA, U.S.A.;

6. Institute of Arctic Biology, University of Alaska Fairbanks, Fairbanks, AK, U.S.A.;

∗ Corresponding author; e-mail: awschiffmiller@alaska.edu.

Keywords: aerial capture, aerial pursuit, facilitated foraging, Exocoetidae, Ommastrephidae, biologging, foraging behavior

Manuscript type: Article.

Abstract

Seabirds are critical ecosystem members providing connectivity between marine and ~~coastal~~ terrestrial ecosystems. Seabird foraging is often facilitated by other marine predators (e.g. tuna) that aggrega~~eting~~ prey at the surface. Some seabirds have been anecdotally reported to 'hawk' (catch mid-air/in-flight) flyingfish~~flying fish~~ and flying squid ~~that are~~ forced into flight by subsurface predators. Hawking avoids water entry and subsequent takeoff, both of~~is less costly than plunge diving,~~ which are known to be~~requires an~~ energy intensive~~expensive take-off to continue foraging~~, but its relative importance in foraging has not been quantified. We analyzed footage from 25 animal-borne video cameras deployed on red-footed boobies (*Sula sula*) at Palmyra Atoll to estimate the frequency of hawking, plunge diving, and 'other' prey capture attempts. Hawking made up 20.3% of all prey capture attempts and had a 39.3% success rate in 2022, but was not observed for the two tracked birds in 2024.~~(39.3% successful).~~ An additional 17 individuals were opportunistically observed in the footage making hawking capture attempts, suggesting hawking is a common behavior, accounting for a greater proportion of foraging effort than previously known. ~~In triaxial accelerometry, hawking appeared more similar to ordinary flying than plunge diving, likely explaining the lack of detection in prior studies.~~ As hawking is only possible when subsurface predators elicit flight escape behaviors of prey, its high frequency suggests such predator facilitation interactions are stronger than currently understood.

Introduction

Seabirds are integral members of pelagic, nearshore, and terrestrial ecosystems and~~serving as a major biological pump and contributing large marine nutrient subsidies to nearshore terrestrial and marine ecosystems [1, 2]. Seabirds are~~ abundant mid and upper trophic level predators, with global estimates in the hundreds of millions of individuals [3]. However, they are also among the most threatened, with nearly half of species in decline, 35% listed as threatened or near-threatened [4], and global seabird abundance likely <30% of historical averages [3]. In the tropics, plunge diving sulids (boobies) are some of the most common seabirds and provide the largest~~highest~~ nitrogen and phosphorus subsidies, key in supporting reef ecosystem health [5, 6], per breeding colony at atolls across the globe [2].

Though known as plunge diving specialists, boobies~~Boobies~~ have been anecdotally observed to pursue, catch, and swallow airborne prey in flight [7–13] (hereafter "hawking" for simplicity). Similar foraging tactics (e.g. "air dipping" [14] and aerial pursuit [15]) are also frequently reported for smaller species, including terns [14, 16, 17]. However, the frequency of hawking behavior, its success rate relative to typical sulid plunge diving tactics, and its overall importance to foraging efficiency is essentially unknown. Hawking avoids~~is likely less~~ energetically costly behaviors associated with~~relative to~~ plunge diving as it does not incur the high-G impact with the water surface nor require~~the energetic expenditure of~~ takeoff to continue foraging~~following a plunge dive~~ [18, 19], and therefore may be an important foraging tactic if common.

Critically, for birds targeting marine prey, hawking is naturally~~likely~~ only possible when foraging is facilitated by other marine predators — such as tuna, dolphins, and sharks — which trap schools of prey against the surface and, in the case of flyingfish~~flying fish~~ (Exocoetidae) and flying squid (Ommastrephidae), elicit their flight escape behavior [20]. So important is this phenomenon that many~~Many~~ tropical seabirds have become near exclusive specialists on flyingfish~~flying fish~~ and squid [14, 20–23] and in some cases are considered nearly 'tuna-obligate' [Maxwell & Morgan 2013, Spear 2007, Au & Pitman 1986]. If seabirds are heavily~~, while plunge diving may still be feasible — if less productive — without subsurface predators, hawking is likely dependent on facilitation interactions. Unfortunately many subsurface predators involved in facilitation have experienced severe declines due to anthropogenic factors [24, 25]. Ongoing fisheries activities (legal and otherwise [26]) in combination with anticipated increases in demand [27] and habitat disruption [24] are expected to increase pressure on marine predator populations. Climate change promises to exacerbate these problems in an assortment of ways [24, 28, 29], including by triggering range shifts [29–33] that may place marine predator populations out of reach of established seabird breeding colonies. Seabirds~~ reliant on hawking and specifically predator facilitation driven hawking, particularly during the breeding season when they are central place foragers, they may ~~may therefore~~ be more vulnerable to climate or fishing-pressure driven shifts in prey and marine predator populations than previously recognized ~~to changes impacting the marine predators responsible for facilitation interactions~~ [17]. Although, especially in more anthropogenically impacted systems, some birds also take advantage of 'vessel facilitation' to capture air-borne prey [Ballance 1997, Muramatsu 2016]

these opportunities are likely less reliable near most breeding locations. If hawking is more common than previously known for some of these species, failing to account for it in estimates of foraging effort could result in a misunderstanding of true foraging effort and an underestimate of the importance of predator facilitation interactions to seabirds.

Biologging may offer a solution to understand the importance of hawking behavior and its relationship to subsurface predators. Biologgers, including accelerometers, temperature-depth recorders (TDRs), and wet-dry sensors, have been used to identify foraging bouts and quantify foraging effort of free-living sulids [9, 11, 37–40]. However, these studies likely miss hawking behavior because it is not readily identifiable from the resulting data. Animal-borne cameras [41, 42] offer an opportunity to address this gap. As cameras have become smaller and more battery efficient [34–36], animal-borne video has become a viable way to observe animal behaviors when direct observations are not possible. Animal-borne video footage can be used to assess the relative frequency and importance of behaviors like hawking, and potentially can be used to identify them in other biologger data~~Accelerometry, in combination with temperature-depth recorders (TDRs) and/or wet-dry sensors, has been used to identify some behaviors of free-living sulids [9, 11, 37–40]. Plunge dives, identified by strong spikes in acceleration, are often used to define foraging bouts and quantify foraging effort. However, without an identified accelerometry signature, these studies likely miss hawking behavior. If hawking is more common than previously known, failing to account for it in accelerometry-based estimates of foraging effort could result in a misunderstanding of true foraging effort and an underestimate of the importance of predator facilitation interactions to seabirds. Combining accelerometry with animal borne cameras [41, 42] offers an opportunity to address this gap, assess the relative importance of the behavior, and potentially identify an accelerometry signal to use~~ in future research.

Red-footed boobies (*Sula sula*; abbreviated "RFBO") are the smallest [43] and most pelagic of boobies [44], and one of the most globally abundant sulids [4]. They are widely reported to forage in association with marine predators and consume primarily flyingfish and flying~~ fish and~~ squid, with many anecdotal reports of in-flight captures [7–12, 20]. Here, we deployed animal-borne cameras, with GPS and accelerometry biologgers, on nesting RFBO at Palmyra Atoll (a small atoll in the Central Tropical Pacific), to estimate the contribution of hawking foraging tactics to overall foraging effort. We show that hawking behavior is quite

common with a high capture success rate for RFBO~~but has a subtle accelerometry signature almost certainly missed in previous analyses~~.

Methods

Data collection. Video cameras, GPS loggers, and accelerometers, were deployed on RFBO at Palmyra Atoll (5.88^{o} N, ~~88, –~~162.07^{o} W~~07~~) in May and November 2022, October 2023, and November 2024. All birds selected for tagging were incubating eggs or brooding young chicks; birds with older chicks were avoided because irregular nest attendance made tag recovery more difficult. Pairs in this nesting stage usually alternate day-long foraging trips with a day tending the nest, though observed foraging trips often ranged up to 2 or 3 days and rarely 4 or more. Only one mate from a pair was captured and tagged. The tracked individual was marked with permanent marker or non-toxic nail polish on their breast and/or the top of their head for easy identification from a distance and to avoid unnecessary disturbance of their mate.

For both logger deployment and retrieval, adult RFBO were caught from their nests by hand or with a telescoping noose pole. Nests less than 2 meters from the ground were considered in range, while nests at greater heights were avoided. During the initial capture all birds were equipped with a unique USGS metal ID band. Mass was recorded for both deployment and retrieval captures by placing birds in a cloth bag and weighing with a spring balance (± 20 g). Equipment deployed on each bird was adjusted to ensure the total mass was no greater than 3% of the bird's mass at deployment [45]. Loggers were attached to feathers with removable adhesive waterproof tape (tesa tape 4651, Hamburg, Germany) and cyanoacrylate glue. Cameras (Table~~Supplemental table~~ 1~~S1~~) were attached facing forward to the central back contour feathers. Accelerometry and accelerometry/GPS, loggers (Table 1)~~Supplemental table S1)~~ were attached to the back behind the camera, directly over the bird's center of mass. Stand-alone GPS units (Table 1)~~Supplemental table S1)~~ were attached to the three central tail feathers. Lighter animals were generally equipped only with GPS and accelerometers or occasionally with cameras and our lightest GPS unit. Cameras were not deployed on birds weighing less than 750 g.

Cameras were programmed to turn on between 10 AM and noon local time the day following deployment to capture foraging behaviors when birds were farthest from the colony and most likely to be actively foraging [37]. Cameras were programmed to record until battery life

was exhausted (roughly 4 hours) in a single day, or to record for 2 hours at the same time each day until the battery was exhausted. After tags were deployed, nests were checked every day until the tagged bird returned from its foraging trip (most often 48 hours after the tags were deployed). Tags were retrieved by recapturing the bird and all tags and tape were removed. Deployments with successful retrievals typically lasted 2-4 days.

Video Annotation & Analysis. Videos were reviewed and annotated using Behavioral Observation Research Interactive Software (BORIS) v.8.20.3 [46]. Each video's unobscured sections were reviewed at half speed or slower at least twice. Annotations were made for a wide suite of behaviors and conditions, and all prey capture attempts (hereafter 'attacks') counted and categorized.

Attacks were categorized as: ' plunge dives', 'hawking', and 'other' (Figure 1, Supplemental table S1~~S2~~, Supplemental videos; SV1, SV2, SV3). Plunge dives were characterized by a rapid flip or downward turn in flight and steep dive into the water from some height. Hawking was characterized by flight low over the water surface in pursuit of airborne prey without landing, regardless of capture success. Instances of near-horizontal water entry from low flight and of dipping dives from sitting on the surface as well as otherwise unclassified attacks were categorized as 'other'. Behaviors such as theft from other birds or scavenging were not counted as attacks. Aborted attacks were ~~also~~ noted separately, though their identification is inherently ~~more~~ subjective. Aborted plunge dives were counted when the bird was deemed~~seemed~~ to have initiated~~initiate~~ a dive but resumed level or ascending flight without reaching the water surface, and aborted hawking counted when the bird soared close to the water surface behind airborne prey, but deemed~~did~~ not ~~seem~~ to have targeted~~target~~ an individual for attempted capture.

Each attack was categorized as successful, failed, or unknown. An attack was counted successful if it was clear the bird had caught prey in its bill, even if swallowing could not be confirmed (Figure 1a~~1d~~, Supplemental videos SV1, SV2). Attacks were counted as failed if prey was not caught (Supplemental video SV1, SV2). Attack success was counted as unknown when footage was obscured by water or feathers and prey capture could not be confirmed or ruled out (Figure 1b~~1e~~, Supplemental videos SV1). Aborted plunge dives and hawking attempts were counted only as 'aborted'~~'aborted'~~.

To calculate attack rate per minute overall and for each attack type, attack counts were divided by the time between the first and last observed attack, including aborted attacks, plus one minute on either end (Supplemental table S2). This was done to exclude periods when birds were commuting between the nest and foraging areas from attack rate calculations. This ‘observed attack window’ included any time when the camera footage was obscured and when birds may have been traveling between foraging bouts.

Attacks made by other untagged birds (individuals not carrying biologging equipment but captured on video by camera-tagged individuals) were noted when enough of the attack was in frame to categorize the type (Figure S1a, Supplemental video SV4) and success was determined in the same manner as for attacks by tagged birds, but these events were not included in calculations for prevalence of attack types or rates as they were observed opportunistically.

~~Accelerometry analysis was performed in R (version 4.5.2). Accelerometry data from plunging and hawking attacks and regular flight, as identified in video footage, were reviewed and representative examples from a single bird selected (Figure 1a, b, c). Static accelerometry was calculated as a 2 second moving window average of the raw accelerometry, and dynamic accelerometry calculated as the raw accelerometry minus the static accelerometry.~~

Results

Video cameras were deployed on 47 individuals at Palmyra Atoll (33 in 2022, 4 in 2023, and 11 in 2024) and 36.7 hours of unobscured footage was recovered and analyzed from 25 individuals. We note that 2023 was an El Niño year with low nest numbers and a large degree of nest failures, and during the 2024 field season the number of nesting birds was still ~~recovering from the 2023 El Niño event, and~~ a tiny fraction of that observed in 2022 and was still recovering from the 2023 El Niño event. Usable footage was not retrieved from any birds in 2023, and from only 2 birds in 2024. A total of 357 completed attacks and 98 aborted attacks made by tagged birds were identified. Total attack counts (excluding aborted attacks) for individuals ranged from 1 to 49 and hawking attacks accounted for 0% to 100% (mean = 19.5%) of observed attacks for any given individual (Figure 3). Total attacks per minute over the observed attack window ranged from 0.16 to 2.06 (mean=0.78) (Supplemental table S2).

Hawking attacks. Of the 25 tagged individuals with attacks captured on camera, 20 made at least one hawking attack (Figure 3), indicating that it is a normal and regular foraging behavior. Hawking attacks accounted for 20.3% of all 2022 attacks (Figure 2). Success or failure of hawking attacks was easy to determine as the field of view remained largely or fully unobscured, and 39.3% were successful (Figure 2, Supplemental video SV2). In addition, 16 hawking attacks (9 successful) made by birds near a camera-carrying bird were opportunistically observed and recorded in 2022 (Supplemental Figure S1a, Supplemental video SV4). In the 2024 footage, no hawking attacks by either camera-tagged bird were observed, despite one having the greatest total number of observed attacks in our dataset (49; Figure 3), but a single successful hawking attack by another RFBO was observed. Hawking attack rate ranged from 0-0.52 (mean = 0.09) attacks per minute over the observed attack window when all birds were included and from 0.03-0.52 (mean = 0.13) when only birds with at least one hawking attack were included.

Plunge diving attacks. Of all attacks in 2022, 64.1% were plunge dives (Figure 2). Success could not be determined and was unknown for the majority of plunge dive attacks (84.5%; Figure 2) as the field of view was usually obscured by feathers, water, and bubbles as the bird impacted, entered, sat on, or exited the water (Supplemental video SV1). Success was confirmed for 13.5%, and failure for only 2.1%, of plunge dives (Figure 2). Between both 2024 birds, 47 plunge diving attacks (4 successful) were observed. Plunge diving attack rate ranged from 0-1.39 (mean = 0.4) attacks per minute.

Other and aborted attacks. The remaining 15.6% of all 2022 attacks were categorized as 'other' (Figure 2). Of these, 55.3% were extremely shallow angle dives (as opposed to plunge dives initiated from a height; Supplemental video SV3). At least one of these appeared to be an attempted hawking attack, but the bird incidentally struck the water as the fish it pursued reentered the water. Underwater dives, starting from a position sitting on the surface, accounted for 34% of 'other' attacks. In 2024, 9 'other' attacks (all unknown success), were counted. Attack rate for 'other' attacks ranged from 0-0.39 (mean = 0.11) attacks per minute.

Eighty (80) aborted attacks were noted in 2022. Of these, 55 (68.75%) were aborted plunge dives and 25 (31.25%) were aborted hawking attacks. In 2024, 16 aborted plunge dives

and 2 aborted hawking attacks were identified. The aborted attack rate ranged from 0-0.65 (mean = 0.17) attacks per minute over the observed attack window. Across all 2022 and 2024 data, 8 instances of scavenging, fighting with con-specifics over food, and theft from con-specifics were observed but not counted as attacks.

Prey. For two of the successful hawking attacks and one of the plunge diving attacks of unknown outcome, the targeted prey was flying squid (family Ommastrephidae; Supplemental Figure S1c,d, Supplemental video SV5). For all remaining attacks where prey type could be identified, the targeted prey was flyingfish~~flying fish~~ (family Exocoetidae, genus unknown). We noted many instances of flyingfish~~flying fish~~ leaving the water in groups of tens to hundreds of individuals. These mass flights usually occurred repeatedly over the course of a several minutes, and typically included evidence of marine predator presence. During these 'fish storms' (minimum n = 6) birds would often make multiple hawking attacks in sequence (Supplemental Figure S1b, Supplemental video SV6). A total of 39 hawking attacks, nearly a third of those observed (19 failed, 9 successful, 11 aborted), made by our tagged birds (as well as 5 made by other birds) were observed in association with 'fish storms'.

~~*Accelerometry*. Paired video and accelerometry data revealed a strong dive signature with spikes in acceleration on all axes as birds dived and hit the water, followed by a period (several seconds to minutes) of low amplitude signals produced by ocean waves while the bird rested at the surface, and finally a rapid oscillation on all axes as birds took off from the water (Figure 1c). This pattern is consistent with previously reported plunge dive accelerometry signatures [9, 47, 48]. In contrast hawking appears in the accelerometry data as a brief, and relatively subtle, disturbance to normal flap-gliding flight (Figure 1b,a).~~

Discussion

Hawking accounted for 20% of attacks observed in animal-borne video footage and was successful 39% of the time, representing a substantial proportion of foraging effort for RFBOs. ~~Although our study did not permit a good estimate of success for plunge dives, others have shown success rates to be high. A 56% success rate was found for masked booby (*Sula*~~

dactylatra) plunge dives (n=9) [41]. For Australasian gannets (*Morus serrator*), Machovsky-Capuska [42, 49] found success rates of 43-45.5% for plunging dives without pursuit and Cansse [50] reported a 57% success rate (with 20% unknown) for plunge-pursuit diving. Though RFBO preform shallower plunge dives [9, 51] and engage in underwater pursuit comparatively rarely [52], their plunge dive success rates are expected to be similarly high. However, even if hawking is less frequently successful than plunge diving, hawking likely accounts for a disproportionately high degree of net energy gain, as plunge dives require energy-expensive takeoffs [18, 19] to continue foraging, regardless of success. In contrast, the energy cost of a failed or aborted hawking attack appears nearly identical to any other low-altitude flight, a common locomotor behavior even when not actively foraging [53]. Though anecdotally reported, hawking behavior has not been accurately accounted for in previous foraging descriptions for sulids. The prevalence of hawking attacks presented here indicates that a large proportion of RFBO foraging effort is not currently being identified using standard biologging analysis methods. For instance using accelerometry, TDRs, and wet/dry activity sensors, Weimerskirch [9] reported 93% of RFBO dives were plunge dives, and 7% surface dives initiated while sitting on the water. For the plunge dives, approximately 30% were described as 'aerial' dives which never entered the water. These proportions are roughly similar to the fraction of plunge dives that struck the water versus those that were aborted in our study. They proposed that the aerial dives "probably correspond to their specific prey capture technique [and] allow red-footed boobies to pursue and capture mobile prey such as flying fishes and squids at or above the surface." However, our video footage and initial observation of associated accelerometry patterns (Figure 1a,b,c) indicates that these previously reported 'aerial' dives were instead most likely aborted plunge dives, while whereas hawking captures, which have a more subtle accelerometry signature, likely went undetected. While a unique and consistent accelerometry signal may be discernible for hawking attacks, no studies have done so to date. The prevalence of hawking attacks presented here indicates that a large proportion of RFBO foraging effort is not currently being identified using standard biologging analysis methods.

Although our study did not permit a good estimate of success for plunge dives, to compare against the success of hawking, others have shown success rates to be high. A 56% success rate was found for masked booby (*Sula dactylatra*) plunge dives (n=9) [41]. For Australasian gannets (*Morus serrator*), Machovsky-Capuska [42, 49] found success rates of 43-45.5% for plunging

dives without pursuit and Cansse [50] reported a 57% success rate (with 20% unknown) for plunge-pursuit diving. Though RFBO perform shallower plunge dives [9, 51] and engage in underwater pursuit comparatively rarely [52], their plunge dive success rates are expected to be similarly high. Plunge diving is an energetically costly method of foraging due to the large amount of time spent in active flight and the necessity of energy-expensive takeoffs [18, 19, green 2009] and regaining altitude to continue foraging, regardless of success. While hawking also requires flight, it does not require a takeoff and likely has a similar energetic cost to any other low-altitude flight, a common locomotor behavior during foraging trips even when not actively foraging [12]. Hawking attacks, whether successful or not, impart almost no opportunity cost as the bird is immediately in position to make another attempt. The relative energetic consequences of hawking and plunge diving foraging tactics remain to be quantified but hawking may increase overall foraging efficiency considerably, even if the energetic cost per unit time is similar or success rate is lower

The numerous documented examples of hawking attacks from the 25 RFBO carrying cameras, and at least 17 other individuals opportunistically observed, demonstrate that this is a common behavior for the Palmyra Atoll population of RFBOs. Data from other studies suggests that hawking is a species-wide behavioral trait and not a population-specific behavior. Dunn et al. [11] reported multiple instances of hawking from a single camera-tagged RFBO, and additional conspecifics caught in frame, nesting in the Chagos archipelago in the Indian Ocean (Figure 3), consistent with other anecdotal reports of RFBO hawking [7, 8, 10, 12].

It is less clear if this behavior is common across all RFBO individuals and conditions. Cameras only recorded a portion of each foraging trip, giving a small window into foraging during the breeding season. We found substantial individual variation in rates of different attack types (Figure 3) indicating individuals may be specializing in foraging tactics [54–56]. Similarly, the relative lack of hawking behavior in 2024, when higher wind speeds were observed and ocean conditions were still relatively poor following the 2023 El Niño event, may be evidence that the use of hawking depends on local wind conditions, changes in prey populations, or changes in subsurface predator populations driven by environmental variability (El Niño, marine heatwaves, ocean warming). The many observations of hawking by individuals unencumbered with tags supports hawking as a normal behavior, not one adopted only in response to the mass or placement of biologgers. Regardless of any differences in attack

behaviors or rates due to individual variation, environmental conditions, or biologger attachment [57], the frequency of observed hawking attacks changes our understanding of foraging effort and behavior for this species. Hawking may also be a more common behavior than realized for other seabird species [13, 16], and foraging effort may be considerably underestimated for them as well. By failing to account for hawking tactics in foraging by RFBO and other seabirds, foraging analyses may underestimate food intake, foraging efficiency, and the strength and importance of interactions between seabirds and other pelagic predators.

Many pelagic seabirds forage in aggregation with subsurface predators [8, 22, 23], increasing the efficiency of foraging by allowing repeated capture attempts and higher success rates within a reduced area and time [58]. Our study strongly suggests such facilitation interactions may be key to unlocking a separate and potentially highly ~~energetically~~ efficient foraging tactic, hawking, for species traditionally considered to be plunge diving specialists. This may require a reexamination of the foraging tactics of pelagic seabirds to quantify the contribution of this critical, but previously unexamined, behavior. While animal -borne cameras are key to documenting and validating it, they still have many challenges: heavy, short battery lives and recording windows, prone to being obscured by feathers and positioning, and requiring extreme effort to analyze and extract data. A critical next step will be using other biologging streams to identify and better understand this behavior. While very similar to skimming flight, a unique and consistent accelerometry signal may ~~still~~ be discernible for hawking attacks; but~~however~~ no studies have described this signal to date. Accurately describing this signal and reassessing previous foraging data should be a priority for future work. It seems likely that marine-predator facilitated foraging will prove to be even more critical to these tropical seabirds than previously acknowledged, potentially changing how we think about what is needed to conserve these species.

Funding. This project was funded by National Science Foundation grants (2113463 to G.A.B, S.A.S, H.Y., and S.M.M.). The Nature Conservancy (TNC) provided cooperative funding through discounted station use rates and station staffing. S.M.M. was partially supported by University of Washington Scholarship, Research and Creative Practice Grants. A.W.S. was also provided support by the Calvin J. Lensink Endowment and UAF's Troth Yeddha Research Assistantship.

Acknowledgments. Thanks to our 2023 and 2024 field techs: Cole Wan Jower and Stella Solasz. Thanks to Morgan Gilmour, Josh Adams, and other 'Palmyra Blue Water Research' team members for training in bird handling and capture methods. Thanks to the US Fish and Wildlife Service Rangers, staff, and volunteers, and TNC station staff, program staff, and volunteers for making the field seasons possible, coordinating travel and accommodations, and generally making the Palmyra field station a great field work experience. Thank you to University of California Santa Barbara undergraduates Ashlyn Foster, Jacob Cain, and Everest Fedler for their many hours of work annotating video footage.

Literature Cited

[1] Otero XL, De La Peña-Lastra S, Pérez-Alberti A, Ferreira TO, Huerta-Diaz MA. 2018 Seabird colonies as important global drivers in the nitrogen and phosphorus cycles. *Nature Communications* **9**, 1, 1–8. (doi:10.1038/s41467-017-02446-8).

[2] Steibl S, Steiger S, Wegmann AS, Holmes ND, Young HS, Carr P, Russell JC. 2024 Atolls are globally important sites for tropical seabirds. *Nature Ecology and Evolution* **8**, 10, 1907–1915. (doi:10.1038/S41559-024-02496-4;SUBJMETA).

[3] Paleczny M, Hammill E, Karpouzi V, Pauly D. 2015 Population Trend of the World's Monitored Seabirds, 1950-2010. *PLOS ONE* **10**, 6, e0129342. (doi:10.1371/journal.pone.0129342).

[4] IUCN. 2025. The IUCN Red List of Threatened Species. Version 2025-2. (accessed on 09 February 2026).

[5] Lange ID, Benkwitt CE. 2024 Seabird nutrients increase coral calcification rates and boost reef carbonate production. *Scientific Reports* **14**, 1, 24937. (doi:10.1038/s41598-024-76759-2).

[6] Dunn RE, Graham NAJ, Jeannot LL, Karkarey R, Gonzalez-Barrios FJ, Lange ID, Fillol JR, Roche R, Stuhr M, Benkwitt CE. 2025 Active and passive pathways of nutrient transfer in coral reef ecosystems. *Coral Reefs* **44**, 4, 1157–1170. (doi:10.1007/s00338-025-02676-z). Diamond A. 1974 The Red-Footed booby on Aldabra Atoll, Indian Ocean. *Ardea* **62**, 196–218.

[7] Ballance LT. 1993 *Community ecology and flight energetics in tropical seabirds of the eastern Pacific: Energetic correlates of guild structure*. Ph.D. thesis, University of California Los Angeles.

[8] Weimerskirch H, Le Corre M, Ropert-Coudert Y, Kato A, Marsac F. 2005 The three-dimensional flight of red-footed boobies: Adaptations to foraging in a tropical environment? *Proceedings of the Royal Society B: Biological Sciences* **272**, 1558, 53–61. (doi: 10.1098/rspb.2004.2918).

[9] Howell SNG. 2014 Why do they fly? In: *The Amazing World of Flyingfish*, pp. 19–24. Princeton University Press.

[10] Dunn RE, Trevail AM, Nicoll MAC, Freeman R, Braman CA, Clark BL, Mitchell C, Schiffmiller AW, Wood H, Votier SC. 2025 Commuting in crosswinds and foraging in fast winds: the foraging ecology of a flying fish specialist. *Proceedings B* **292**, 2052. (doi: 10.1098/RSPB.2025.0774).

[11] Muramatsu K, Yamamoto J, Abe T, Nishizawa B, Hoshi N, Ohwada M, Watanuki Y, Sakurai Y. 2016 A red-footed booby catching airborne squid. *Journal of the Yamashina Institute for Ornithology* **47**, 2, 130–135. (doi:10.3312/JYIO.47.130).

[12] Lerma M, Castillo-Guerrero JA, Hernández-Vázquez S, Garthe S. 2020 Foraging ecology of a marine top predator in the Eastern Tropical Pacific over 3 years with different ENSO phases. *Marine Biology* **167**, 7, 88. (doi:10.1007/s00227-020-03699-6).

[13] Ashmole NP, Ashmole MJ. 1967 Comparative feeding ecology of sea birds of a tropical oceanic island. *Bulletin of the Peabody Museum of Natural History* **40**, 1, 1–131.

[14] Hernández J, Arroyo JI. 2025 Foraging guilds of seabirds. *Ornithology Research* **33**, 1, 57. (doi:10.1007/s43388-025-00265-w). Tiongson AJC. 2021 A common tern sterna hirundo capturing an airborne flying squid: A novel foraging technique? *Marine Ornithology* **49**, 2, 253–255.

[15] Maxwell SM, Morgan LE. 2013 Foraging of seabirds on pelagic fishes: Implications for management of pelagic marine protected areas. *Marine Ecology Progress Series* **481**, 289–303. (doi:10.3354/MEPS10255).

[16] Shaffer SA, Costa DP, Weimerskirch H. 2001 Behavioural factors affecting foraging effort of breeding wandering albatrosses. *Journal of Animal Ecology* **70**, 5, 864–874.

[17] Green JA, White CR, Bunce A, Frappell PB, Butler PJ. 2009 Energetic consequences of plunge diving in gannets. *Endangered Species Research* **10**, 1, 269–279. (doi:10.3354/ESR00223).

[18] Spear LB, Ainley DG, Walker WA. 2007 Foraging Dynamics of Seabirds in the Eastern Tropical Pacific Ocean **35**, 1–99.

[19] Kaschner K, Karpouzi V, Watson R, Pauly D. 2006 Forage fish consumption by marine mammals and seabirds, p. 33- 46. In: Alder, J., Pauly, D., (eds). On the multiple uses of forage fish: from ecosystems to markets. *Fisheries Centre Research Reports* **14**, 3, 33–46.

[20] Hebshi AJ, Duffy DC, Hyrenbach KD. 2008 Associations between seabirds and subsurface predators around Oahu, Hawaii. *Aquatic Biology* **4**, 1, 89–98. (doi:10.3354/AB00098).

[21] Au DWK, Pitman RL. 1986 Seabird Interactions with Dolphins and Tuna in the Eastern Tropical Pacific. *The Condor* **88**, 3, 304–317. (doi:10.2307/1368877).

[22] McCauley DJ, Pinsky ML, Palumbi SR, Estes JA, Joyce FH, Warner RR. 2015 Marine defaunation: Animal loss in the global ocean. *Science* **347**, 6219, 1255641. (doi:10.1126/science. 1255641).

[23] Fortuna CM, Fortibuoni T, Bueno-Pardo J, Coll M, Franco A, Giménez J, Stranga Y, Peck MA, Claver C, Brasseur S, *et al.* 2024 Top predator status and trends: ecological implications, monitoring and mitigation strategies to promote ecosystem-based management. *Frontiers in Marine Science* **11**. (doi:10.3389/fmars.2024.1282091).

[24] Khan AMA, Jiang Mg, Yang Xq, Apriliani IM, Purba NP, Wiryawan B, Taurusman AA, Pasaribu B. 2024 Illegal fishing threatens the sustainability of future tuna commodities in Indonesia. *Marine Policy* **159**, 105936. (doi:10.1016/j.marpol.2023.105936).

[25] Kawamoto T. 2022 A challenge to estimate global canned tuna demand and its impact on future tuna resource management using the gamma model. *Marine Policy* **139**, 105016. (doi: 10.1016/j.marpol.2022.105016).

[26] Braun CD, Lezama-Ochoa N, Farchadi N, Arostegui MC, Alexander M, Allyn A, Bograd SJ, Brodie S, Crear DP, Curtis TH, *et al.* 2023 Widespread habitat loss and redistribution of marine top predators in a changing ocean. *Science Advances* **9**, 32, eadi2718. (doi:10.1126/sciadv.adi2718).

[27] Erauskin-Extramiana M, Chust G, Arrizabalaga H, Cheung WWL, Santiago J, Merino G, Fernandes-Salvador JA. 2023 Implications for the global tuna fishing industry of climate change-driven alterations in productivity and body sizes. *Global and Planetary Change* **222**, 104055. (doi:10.1016/j.gloplacha.2023.104055).

[28] Lehodey P, Senina I, Calmettes B, Hampton J, Nicol S. 2013 Modelling the impact of climate change on Pacific skipjack tuna population and fisheries. *Climatic Change* **119**, 1, 95–109. (doi:10.1007/s10584-012-0595-1).

[29] Silva PM, Borges FO, Pita C, Costa JL, Teixeira CM. 2025 Projecting climate change impacts on the distribution of the most captured tuna species on Atlantic waters. *Biodiversity and Conservation* **34**, 10, 3633–3656. (doi:10.1007/s10531-025-03121-x).

[30] Ganachaud A, Sen Gupta A, Brown JN, Evans K, Maes C, Muir LC, Graham FS. 2013 Projected changes in the tropical Pacific Ocean of importance to tuna fisheries. *Climatic Change* **119**, 1, 163–179. (doi:10.1007/s10584-012-0631-1).

[31] Nicol S, Lehodey P, Senina I, Bromhead D, Frommel AY, Hampton J, Havenhand J, Margulies D, Munday PL, Scholey V, *et al.* 2022 Ocean Futures for the World's Largest Yellowfin Tuna Population Under the Combined Effects of Ocean Warming and Acidification. *Frontiers in Marine Science* **9**. (doi:10.3389/fmars.2022.816772).

[32] Watanabe YY, Takahashi A. 2013 Linking animal-borne video to accelerometers reveals prey capture variability. *Proceedings of the National Academy of Sciences of the United States of America* **110**, 6, 2199–2204. (doi:10.1073/PNAS.1216244110).

[33] Michel L, Cianchetti-Benedetti M, Catoni C, Dell'Omo G. 2022 How shearwaters prey. New insights in foraging behaviour and marine foraging associations using bird-borne video cameras. *Marine Biology* **169**, 1. (doi:10.1007/S00227-021-03994-W).

[34] Moll RJ, Millspaugh JJ, Beringer J, Sartwell J, He Z. 2007 A new 'view' of ecology and conservation through animal-borne video systems. *Trends in Ecology & Evolution* **22**, 12, 660–668. (doi:10.1016/j.tree.2007.09.007).

[35] Weimerskirch H, Le Corre M, Jaquemet S, Marsac F. 2005 Foraging strategy of a tropical seabird, the red-footed booby, in a dynamic marine environment. *Marine Ecology Progress Series* **288**, 251–261. (doi:10.3354/MEPS288251).

[36] van Oordt F, Silva J, Patterson A, Elliott KH. 2024 Plunge-diving into dynamic body acceleration and energy expenditure in the Peruvian booby. *The Journal of experimental biology* **227**, 22. (doi:10.1242/JEB.249555).

[37] Roper T-Couder T Y, Grémillet D, Ryan P, Kato A, Naito Y, Le Maho Y. 2004 Between air and water: the plunge dive of the Cape Gannet Morus capensis. *Ibis* **146**, 281–290. (doi: 10.1111/j.1474-919x.2003.00250.x).

[38] Sommerfeld J, Kato A, Ropert-Coudert Y, Garthe S, Hindell MA. 2013 Foraging Parameters Influencing the Detection and Interpretation of Area-Restricted Search Behaviour in Marine Predators: A Case Study with the Masked Booby. *PLOS ONE* **8**, 5, e63742. (doi:10.1371/ JOURNAL.PONE.0063742).

[39] Machovsky-Capuska GE, Priddel D, Leong PH, Jones P, Carlile N, Shannon L, Portelli D, McEwan A, Chaves AV, Raubenheimer D. 2016 Coupling bio-logging with nutritional

geometry to reveal novel insights into the foraging behaviour of a plunge-diving marine predator. *New Zealand Journal of Marine and Freshwater Research* **50**, 3, 418–432. (doi: 10.1080/00288330.2016.1152981).

[40] Machovsky-Capuska GE, Vaughn RL, Würsig B, Katzir G, Raubenheimer D. 2011 Dive strategies and foraging effort in the Australasian gannet Morus serrator revealed by underwater videography. *Marine Ecology Progress Series* **442**, 255–261. (doi:10.3354/MEPS09458).

[41] Van Oordt F, Torres-Mura JC, Hertel F. 2018 Ecomorphology and foraging behaviour of Pacific boobies. *Ibis* **160**, 2, 313–326. (doi:10.1111/ibi.12545).

[42] Young HS, Shaffer SA, McCauley DJ, Foley DG, Dirzo R, Block BA. 2010 Resource partitioning by species but not sex in sympatric boobies in the central Pacific Ocean. *Marine Ecology Progress Series* **403**, 291–301. (doi:10.3354/meps08478).

[43] Vandenabeele SP, Shepard EL, Grogan A, Wilson RP. 2012 When three per cent may not be three per cent; device-equipped seabirds experience variable flight~~Xight~~ constraints. *Mar Biol* **159**, 1–14. (doi:10.1007/s00227-011-1784-6).

[44] Friard O, Gamba M. 2016 BORIS: a free, versatile open-source event-logging software for video/audio coding and live observations. *Methods in ecology and evolution* **7**, 11, 1325–1330. (doi:10.1111/2041-210X.12584).

[45] Sharker SI, Holekamp S, Mansoor MM, Fish FE, Truscott TT. 2019 Water entry impact dynamics of diving birds. *Bioinspiration & Biomimetics* **14**, 5, 056013. (doi:10.1088/1748-3190/ ab38cc).

[46] Ropert-Coudert Y, Grémillet D, Kato A, Ryan PG, Naito Y, Le Maho Y. 2004 A fine-scale time budget of Cape gannets provides insights into the foraging strategies of coastal seabirds. *Animal Behaviour* **67**, 5, 985–992. (doi:10.1016/j.anbehav.2003.09.010).

[47] Machovsky-Capuska GE, Howland HC, Raubenheimer D, Vaughn-Hirshorn R, Würsig B, Hauber ME, Katzir G. 2012 Visual accommodation and active pursuit of prey underwater in a plunge-diving bird: the Australasian gannet. *Proceedings of the Royal Society B: Biological Sciences* **279**, 1745, 4118–4125. (doi:10.1098/rspb.2012.1519).

[48] Cansse T, Fauchet L, Wells M, Arnould J. 2020 Factors influencing prey capture success and profitability in Australasian gannets (Morus serrator). *Biology Open* **9**, bio.047514. (doi: 10.1242/bio.047514).

[49] Lewis S, Schreiber EA, Daunt F, Schenk GA, Orr K, Adams A, Wanless S, Hamer KC. 2005 Sex-specific foraging behaviour in tropical boobies: Does size matter? *Ibis* **147**, 2, 408–414. (doi:10.1111/J.1474-919X.2005.00428.X).

[50] Corre ML. 1997 Diving Depths of Two Tropical Pelecaniformes: The Red-Tailed Tropicbird and the Red-Footed Booby. *The Condor* **99**, 4, 1004. (doi:10.2307/1370157).

[51] Ballance LT, Pitman RL, Reilly SB. 1997 Seabird community structure along a productivity gradient: Importance of competition and energetic constraint. *Ecology* **78**, 5, 1502–1518. (doi:10.1890/0012-9658(1997)078[1502:SCSAAP]2.0.CO;2).

[52] Ceia FR, Ramos JA. 2015 Individual specialization in the foraging and feeding strategies of seabirds: a review. *Marine Biology* **162**, 10, 1923–1938. (doi:10.1007/s00227-015-2735-4).

[53] Newsome SD, Tinker MT, Gill VA, Hoyt ZN, Doroff A, Nichol L, Bodkin JL. 2015 The interaction of intraspecific competition and habitat on individual diet specialization: a near range-wide examination of sea otters. *Oecologia* **178**, 1, 45–59. (doi:10.1007/s00442-015-3223-8).

[54] Weimerskirch H, Corre ML, Ropert-Coudert Y, Kato A, Marsac F. 2006 Sex-specific foraging behaviour in a seabird with reversed sexual dimorphism: the red-footed booby. *Oecologia* **146**, 4, 681–691. (doi:10.1007/s00442-005-0226-x).

[55] Webster MM, Rutz C. 2020 How STRANGE are your study animals? *Nature* **582**, 7812, 337–340. (doi:10.1038/d41586-020-01751-5).

[56] Thiebault A, Semeria M, Lett C, Tremblay Y. 2016 How to capture fish in a school? Effect of successive predator attacks on seabird feeding success. *Journal of Animal Ecology* **85**, 1, 157–167. (doi:10.1111/1365-2656.12455).

Tables

Table 1: Biologger details: types, settings, attachment points

**Logger Type**	**Manufacturer: model**	**Mass (g)**	**Settings**	**Attachment location**
Camera	Little Leonardo Inc.: DVL400M	15	30 fps	central back contour feathers
Accelerometer	Technosmart: Axy 5XS	3	25 Hz 8 Gs	central back contour feathers. Behind camera, directly over the bird's center of mass
Accelerometer + GPS	Technosmart: AxyTrek Mini	7.5	GPS: 30 s fix rate<br>Axy: 25 Hz, 8 Gs	central back contour feathers. Behind camera, directly over the bird's center of mass
GPS	Catlog: Gen2	12	30 s fix rate	Central 3 tail feathers
GPS	Lotek: Pinpoint240	7.5	1 min fix rate	Central 3 tail feathers

Figures

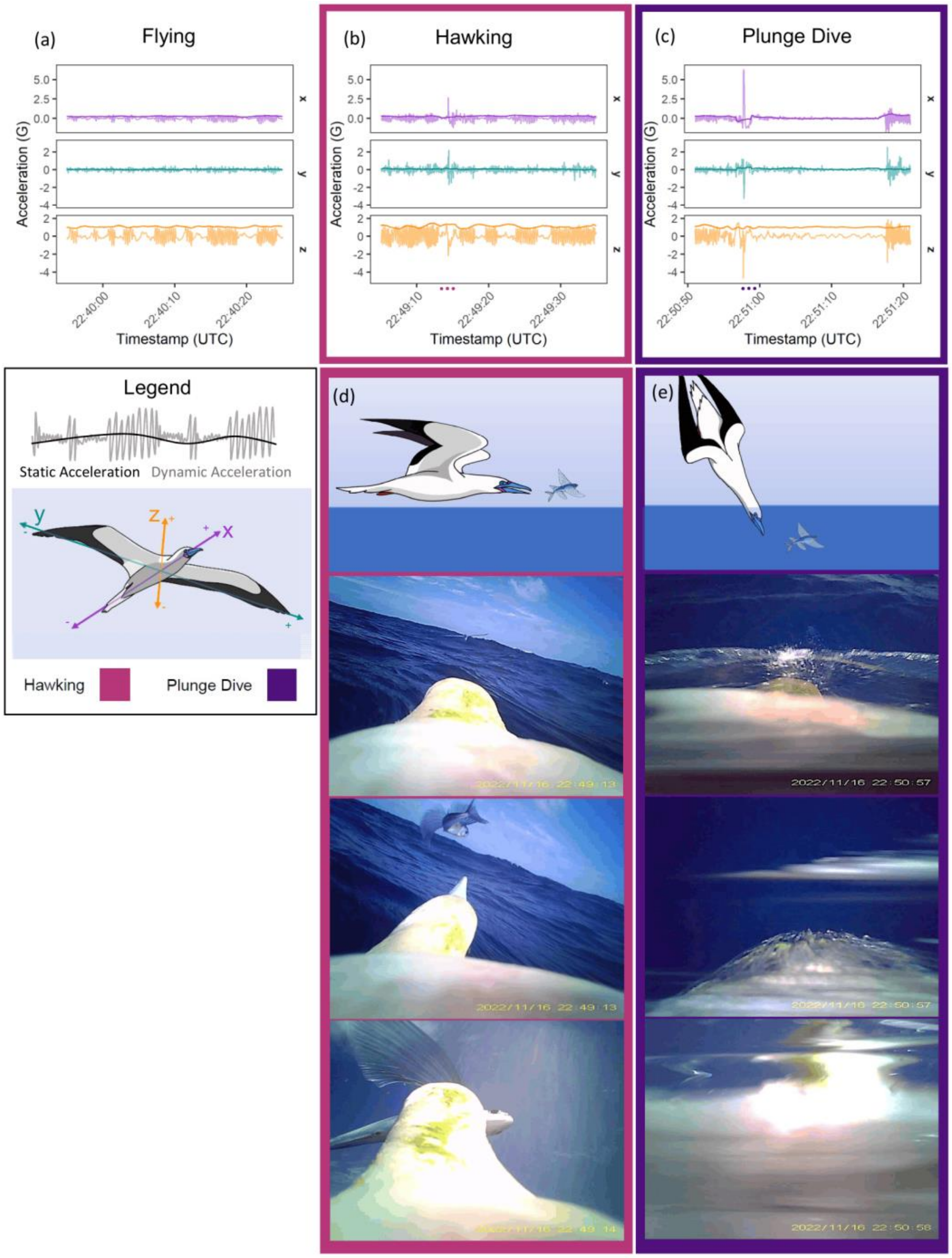

(a)
Flying
(b)
Hawking
(c)
Plunge Dive
Acceleration (G)
x
y
z
5.0
2.5
0.0
2
0
-2
-4
22:40:00
22:40:10
22:40:20
22:49:10
22:49:20
22:49:30
22:50:50
22:51:00
22:51:10
22:51:20
Timestamp (UTC)
Legend
Static Acceleration
Dynamic Acceleration
Hawking
Plunge Dive
(d)
(e)
2022/11/16 22:49:13
2022/11/16 22:49:13
2022/11/16 22:49:14
2022/11/16 22:50:57
2022/11/16 22:50:57
2022/11/16 22:50:58

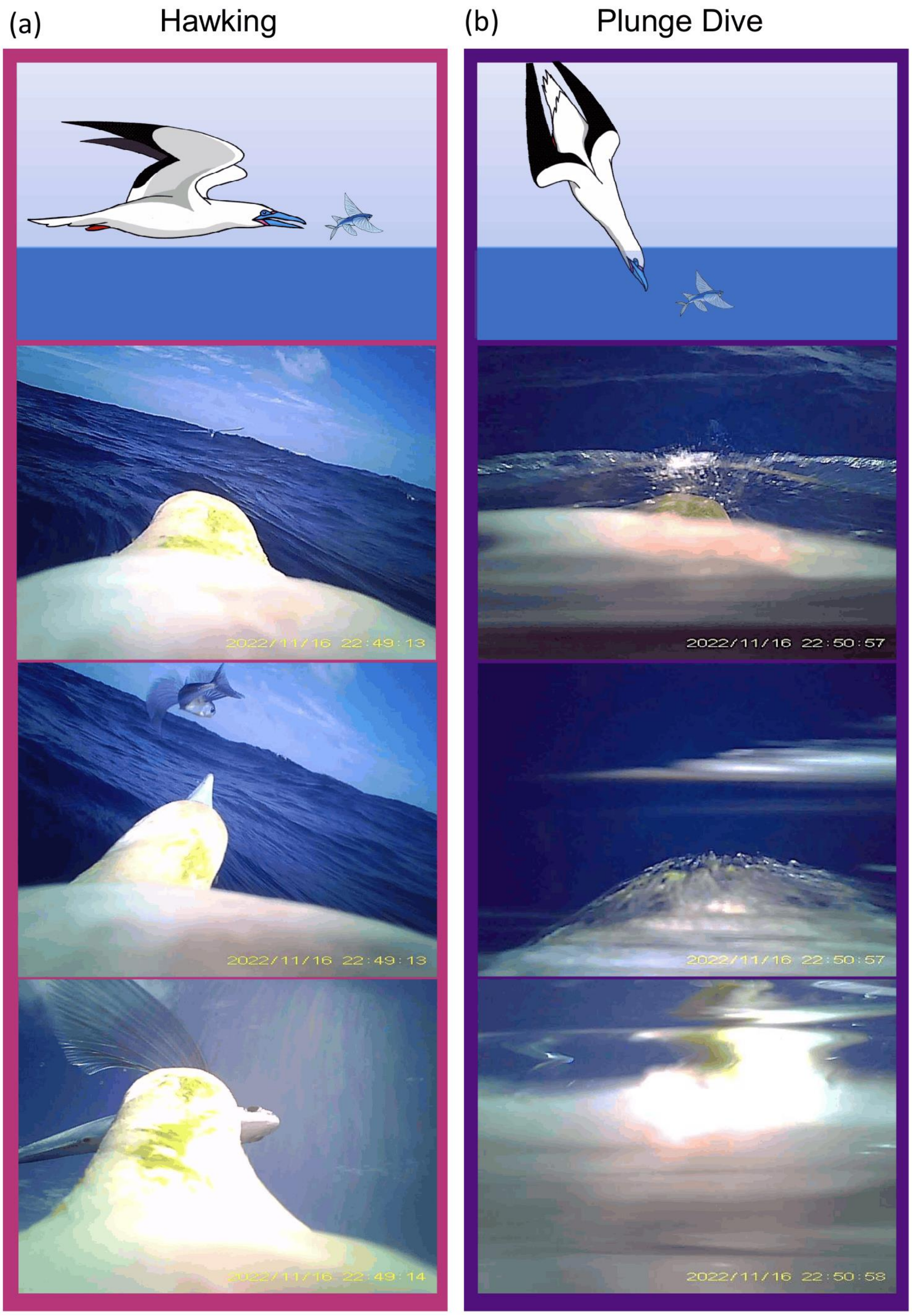

Figure 1: Examples of hawking and plunge diving from video footage.~~ and accelerometry (25 Hz).~~ A) ~~30 s of~~

~~accelerometry from low altitude flapping and soaring flight. B) 30 s of accelerometry from a successful hawking attack. C) 30 s of accelerometry from a plunge dive, success unknown, followed by sitting at the water surface and takeoff. Static acceleration is the 2s moving average of raw accelerometry. Dynamic acceleration is the raw acceleration minus the static acceleration. Static acceleration on the z axis is centered on 1, accounting for gravitational acceleration. D)~~ Diagram ~~of hawking attack~~ and sequence of 3 still images from video footage of hawking attack (successful). B) ~~matching B). E)~~ Diagram ~~of plunge dive attack~~ and sequence of 3 still images from video footage of plunge dive attack (success unknown~~matching C~~). Time stamps in UTC (local time UTC -10). See Supplemental videos SV1 and SV2).

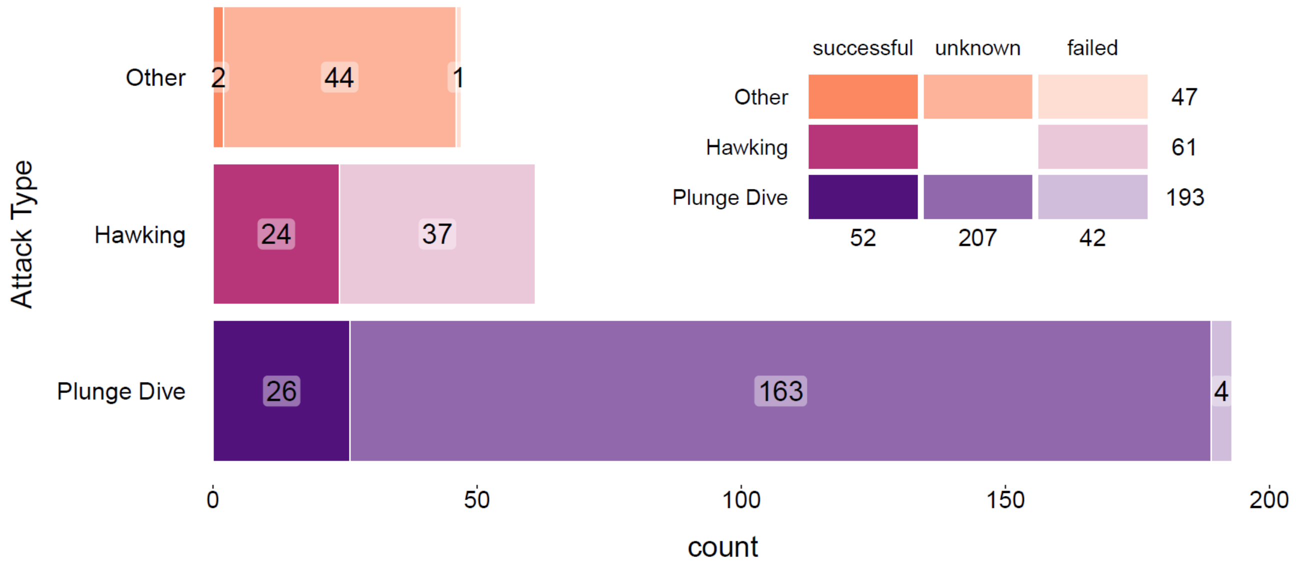

Figure 32: Attack type and success counts, 2022. No hawking attacks had unknown success. Aborted plunge and hawking attacks are not included. Opportunistically observed attacks made by other individuals are not included.

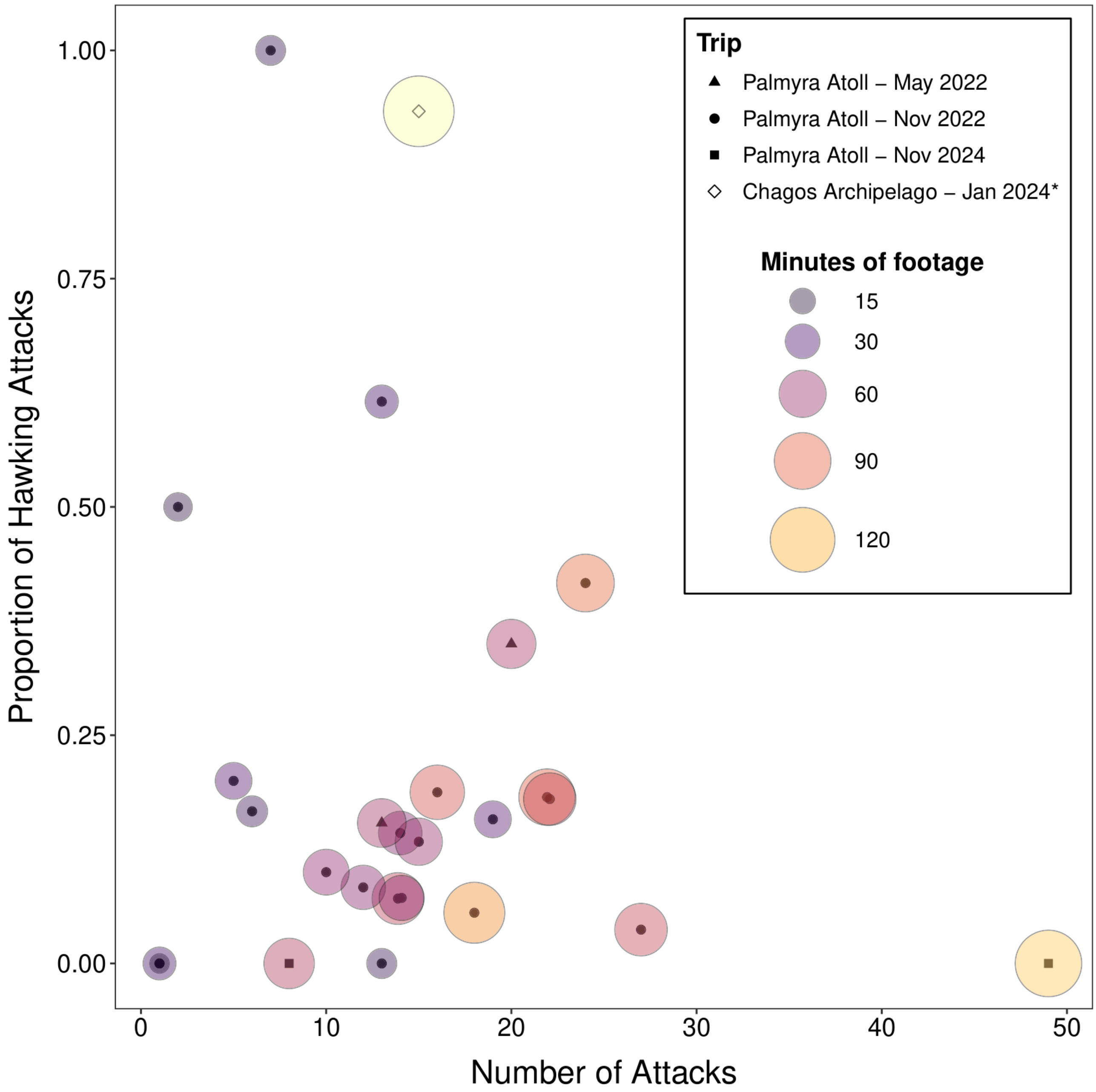

Figure 23: High variation in proportion of hawking, number of observed attacks, and total minutes of unobscured footage per bird. Palmyra Atoll November 2024 birds not included in counts in Figure 2 3 (see discussion). * Chagos Archipelago data as reported by [11]
