# Supplementary material for: Unrecognized importance of hawking prey capture tactics in a tropical plunge-diving seabird
Source: Biol Lett. 2026 Nov 4;22(11):20260292. doi: 10.1098/rsbl.2026.0292 (PMC13730644; doi:10.1098/rsbl.2026.0292)
Supplement: Supplementary file 1 [file rsbl.2026.0292.zip › rsbl.2026.0292/rsbl20260292_si_002.pdf]

Unrecognized importance of hawking prey capture tactics in a tropical plunge diving seabird: Supplemental Material

Abigail Schiffmiller[1*], Charles A. Braman[2], Michael Johns[3], Sara M. Maxwell[4], Hillary Young[2], Scott A. Shaffer[5], Greg A. Breed[1,6]

August 28, 2026

S1 Supplemental Tables

Table S1: Prey Capture Attempt (attack) types

**Attack type**	**Description**	**Example(s)**
Plunge dive	Steep plunge dive out of the air into the water, with the bird becoming fully submerged before resurfacing (type 1) or dive striking the water's surface where the bird never became fully submerged (type 2)	Fig. 1e, Supp. video SV1
Hawking	bird pursued an airborne prey item or snatched an item at the water surface while flying and without landing on or entering the water	Fig. 1d, Supp. Fig. 1, Supp. videosSV2, SV4, SV5, SV6
Other	attacks that could not be otherwise identified as plunge dives or hawking including: dives from sitting on the surface, very shallow angle water entries	Supp. video SV3
Aborted	plunge dive where bird resumes level or ascending flight without reaching water surface, or pursuit of prey as if hawking but without approaching close enough or where prey is captured by another individual instead	Supp. video SV6

S2 Supplemental Figures

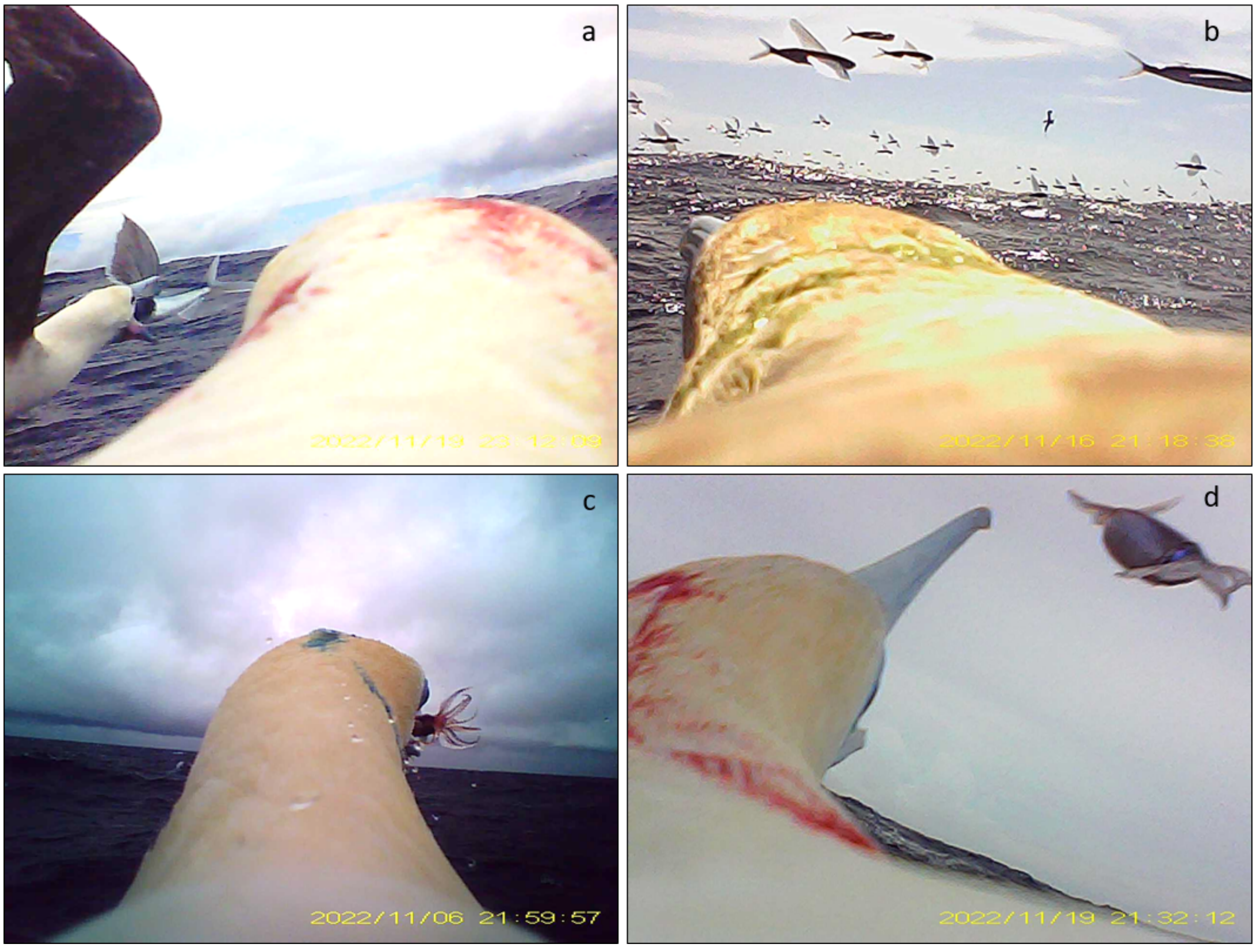

Figure S1: Examples from video footage. A) Example of hawking pursuit and capture of flying fish by an un-tagged RFBO (Supplemental Video SV4). B) Example screenshot from video of an RFBO flying through a school of flying fish. During this sequence, this bird made 3 successful hawking attacks (Supplemental Video SV6). C) and D) Examples of hawking pursuit and capture of flying squid (Supplemental Video SV5). Time stamps in UTC, local time is UTC -10.

S3 Supplemental Videos

Video SV1: Supplemental video: Plunge dives. Examples of plunge dive footage at full speed and 0.5x speed; unknown success plunge dive corresponding with images from Figure 1a, successful plunge dive (to surface), failed plunge dive

Video SV2: Supplemental video: Hawking. Examples of hawking attacks by tagged birds at full and 0.5x speed; successful hawking corresponding with images from Figure 1b, another successful hawking example, failed hawking example

Video SV3: Supplemental video: Other. Examples of attacks classified as 'other': water entry from near horizontal posture near surface, dipping under water from seated on surface.

Video SV4: Supplemental video: Other birds hawking. Examples of other birds, not the camera carrying bird, making hawking prey capture attempts (variably slowed to show event): Successful hawking corresponding to supplemental figure 1a, two additional successful hawking examples, one failed hawking example

Video SV5: Supplemental video: Hawking flying squid. Two examples of RFBO hawking flying squid (both successful), at full speed and 0.5x speed.

Video SV6: Supplemental video: Fish Storms. Example of a 'fish storm': when large numbers of flying fish are forced into flight by subsurface predators. Short example slowed to 0.5x and 0.25x speed, followed by 5 minutes of repeated 'fish storm clouds' during which the bird made multiple hawking attempts (3 successful).
